# Supplementary material for: Complexome profiling of the Chlamydomonas psb28 mutant reveals TEF5 as an early PSII assembly factor
Source: Plant Cell. 2025 Mar 17;37(6):koaf055. doi: 10.1093/plcell/koaf055 (PMC12164590; doi:10.1093/plcell/koaf055)
Supplement: koaf055_Supplementary_Data [file koaf055_supplementary_data.zip › psb28_interactive.zip!/plots/intensity/qualityPlots/peptides/_Cre12.g485200.t1.1.html]

### Cre12.g485200.t1.1

Trivial name:   
SeqCoverage [median]: 10.00  
Mass: 23.17 kDa  
  
 

| Sequence | MissedCleavage | Mass [Da] | wtIntensity | muIntensity | Score |
| --- | --- | --- | --- | --- | --- |
| MALEAGVMAITVEFAQDLNNK | 0 | 2264.12 | 18447.65 | 18474.22 | 45.35 |
|  |  |  | 18447.6 | 18474.2 |  |
